# Scoring Assessments in Stevens-Johnson Syndrome and Toxic Epidermal Necrolysis

**DOI:** 10.3389/fmed.2022.883121

**Published:** 2022-06-16

**Authors:** Allison S. Dobry, Sonia Himed, Margo Waters, Benjamin H. Kaffenberger

**Affiliations:** ^1^Department of Dermatology, University of California, San Francisco, San Francisco, CA, United States; ^2^Division of Dermatology, Ohio State University Wexner Medical Center, Columbus, OH, United States; ^3^Ohio State University College of Medicine, Columbus, OH, United States

**Keywords:** SJS/TEN, scoring assessment, drug reaction, epidermal necrolysis, dermatology

## Abstract

Epidermal necrolysis, the unifying term for Stevens-Johnson syndrome (SJS) and toxic epidermal necrolysis (TEN), is a severe cutaneous drug reaction associated with high morbidity and mortality. Given the rarity of this disease, large-scale prospective research studies are limited. Significant institutional and geographical variations in treatment practices highlight the need for standardization of clinical assessment scores and prioritization of research outcome measures in epidermal necrolysis. At the present, clinical assessment is typically simplified to total body surface area (BSA) involvement, with little focus on morphology. Validated clinical scoring systems are used as mortality prognostication tools, with SCORTEN being the best-validated tool thus far, although the ABCD-10 has also been recently introduced. These tools are imperfect in that they tend to either overestimate or underestimate mortality in certain populations and are not designed to monitor disease progression. Although mortality is often used as a primary endpoint for epidermal necrolysis studies, this outcome fails to capture more nuanced changes in skin disease such as arrest of disease progression while also lacking a validated skin-directed inclusion criterion to stratify patients based on the severity of skin disease at study entry. In addition to mortality, many studies also use BSA stabilization or time to re-epithelialization as endpoints, although these are not clearly defined morphologically, and inter- and intra-rater reliability are unclear. More specific, validated cutaneous assessment scores are necessary in order advance therapeutic options for epidermal necrolysis. In this review, we summarize the strengths and weaknesses of current clinical assessment practices in epidermal necrolysis and highlight the need for standardized research tools to monitor cutaneous involvement throughout the hospitalization.

## Introduction

Epidermal necrolysis, the unifying term for Stevens-Johnson syndrome (SJS) and toxic epidermal necrolysis (TEN), is a severe cutaneous drug reaction associated with high morbidity and mortality (1–3). It is considered to be the most life-threatening dermatologic disease with a mortality incidence of 15% overall, and up to 50% in the elderly (4, 5). Increasing recognition is also being given to the long-term multisystem sequelae of epidermal necrosis present in the majority of survivors, including permanent mucosal damage, cutaneous dyspigmentation and scarring, and resultant mental illness (5). Despite its severity, epidermal necrosis has no FDA-approved therapeutics in use. Treatment, including no treatment, varies significantly by physician specialty, institutional geography, and institutional experiences. In this review, we summarize the strengths and weaknesses of current clinical assessment practices epidermal necrolysis and highlight the need for standardized research tools to monitor cutaneous involvement throughout hospitalization. More specific, validated cutaneous assessment scores are necessary to appropriately risk-stratify patients on study entry, assess skin disease change in response to treatment, and ultimately advance therapeutic options for epidermal necrolysis.

## Strengths and Weaknesses of Scorten

### The Creation of SCORTEN and External Validation

The severity-of-illness score for TEN (SCORTEN) is a mortality prognostication tool for epidermal necrolysis (1). It was developed in 2000 by a team in France, using 165 patients to identify significant variables via a logistic regression model and 75 patients to internally validate the results (1). From this model, the researchers identified seven equally weighted parameters that are risk factors for death: age >40 years, malignancy, heart rate >120 beats per minute, initial percentage of epidermal detachment >10%, serum urea >10 mmol/L, serum glucose >14 mmol/L, and bicarbonate <20 mmol/L (score range: 0–7, Table 1). Collectively, these comprise the SCORTEN, which can predict risk of mortality ranging from 3.2 to 90.0%. Originally, this score was meant to be calculated once within 24 h of admission. Despite this initial intent, authors from this group later published an analysis that demonstrated SCORTEN performance on the first 5 days of hospitalization remained high (and performed even better on day 3), and thus recommended SCORTEN calculation on both days 1 and 3 (6).

**Table 1:** Comparison of mortality prognostic tools ABCD-10 and SCORTEN.

**ABCD-10**		**SCORTEN**	
Age >50 years old	1 point	Age >40 years old	1 point
Bicarbonate <20 mmol/L	1 point	Malignancy	1 point
Cancer/Malignancy	2 points	Heart Rate >120 beats per minute	1 point
Dialysis prior to admission	3 points	Initial Epidermal Detachment BSA >10%	1 point
Initial Epidermal Detachment BSA ≥10%	1 point	Serum urea >10 mmol/L	1 point
		Serum glucose >14 mmol/L	1 point
		Bicarbonate <20 mmol/L	1 point
**Score Range**: 0–8		**Score Range**: 0–7

*A SCORTEN score of 0–1 predicts a mortality rate of 3.2%, a score of 2 as 12.1%, score of 3 as 35.3%, a score of 4 and 54.3 and a score ≥5 as 90%*.

*An ABCD-10 score of 0 predicts a mortality rate of 2.3%, a score of 1 as 5.4%, a score of 2 as 12.3%, a score of 3 as 25.5%, a score of 4 as 45.7, a score of 5 as 67.4 and a score of 6 as 83.6*.

In the two decades following its conception, SCORTEN has been widely used and validated in patient populations around the world. In an effort to summarize its use over the past two decades, a group of researchers performed a meta-analysis to better understand the accuracy of SCORTEN in predicting mortality (7). Overall, 64 studies were included. SCORTEN was found to be an overall good predictor of mortality but tends to underestimate mortality for values <3 and overestimate for values >3. Certain factors were associated with reduced predictive accuracy, such as mean age of patients and ending year of the study. SCORTEN tended to underestimate mortality in older cohorts of patients and overestimate mortality in more recent studies. BSA involvement may influence SCORTEN predictiveness, although the results are more varied. One study found that SCORTEN underestimated mortality for a cohort of patients with TEN (BSA > 30%) (8), but another study found SCORTEN retained good predictive ability in burn center patients (9).

### Critiques of SCORTEN and Attempts at Modified SCORTEN Models

Perhaps the most common criticism of SCORTEN is that it simplifies continuous and dynamic biologic measurements into dichotomous variables, thereby losing a significant amount of information in the process, particularly in the skin assessment which does not regard morphology or locations. Additionally, SCORTEN was originally meant to be used at a single timepoint rather than as a daily monitoring tool. Interestingly some studies have found that either delayed or sequential use of SCORTEN provides improved prognostication (6, 10). Another common concern is that defining BSA remains somewhat subjective, and may vary from one provider to another depending on how BSA involvement is estimated and whether the provider measures only desquamated skin vs. skin with bullae.

In response to this, a group of researchers designed a refined model from 369 patients in the RegiSCAR study that they termed the auxiliary score which scores both age and BSA differently (11). The auxiliary score divides age into three groups (31–55, 56–75, and ≥75 years). The score additionally uses a higher cutoff to differentiate between BSA involvement at >30%. Some studies have found that models that differentiate between BSA >30%, as in TEN, may have better prognostic ability (8, 10, 11). However, authors of the auxiliary score concluded that SCORTEN should remain the model of choice in the clinical setting, whereas the auxiliary score may be useful in retrospective research with missing biochemical data.

The role of other biochemical markers in predicting mortality risk has also been investigated. A group recently found that the ratio of red cell distribution width to hemoglobin (RDW/Hb) is predictive of mortality (12). They incorporated this value into the SCORTEN and named this new model the Re-SCORTEN. Overall, they found improved mortality prognostication with this revised model as compared to SCORTEN alone, but this scoring model has not yet been validated in other populations.

Despite these critiques, SCORTEN has remained the gold standard for not only predicting patient mortality, but is also frequently used in study outcomes to compare therapy efficacy by survival to expected mortality, as well as compare quality of care between institutions (13, 14).

## Strengths and Weaknesses of ABCD-10

### The Creation of ABCD-10

**Figure 1:**
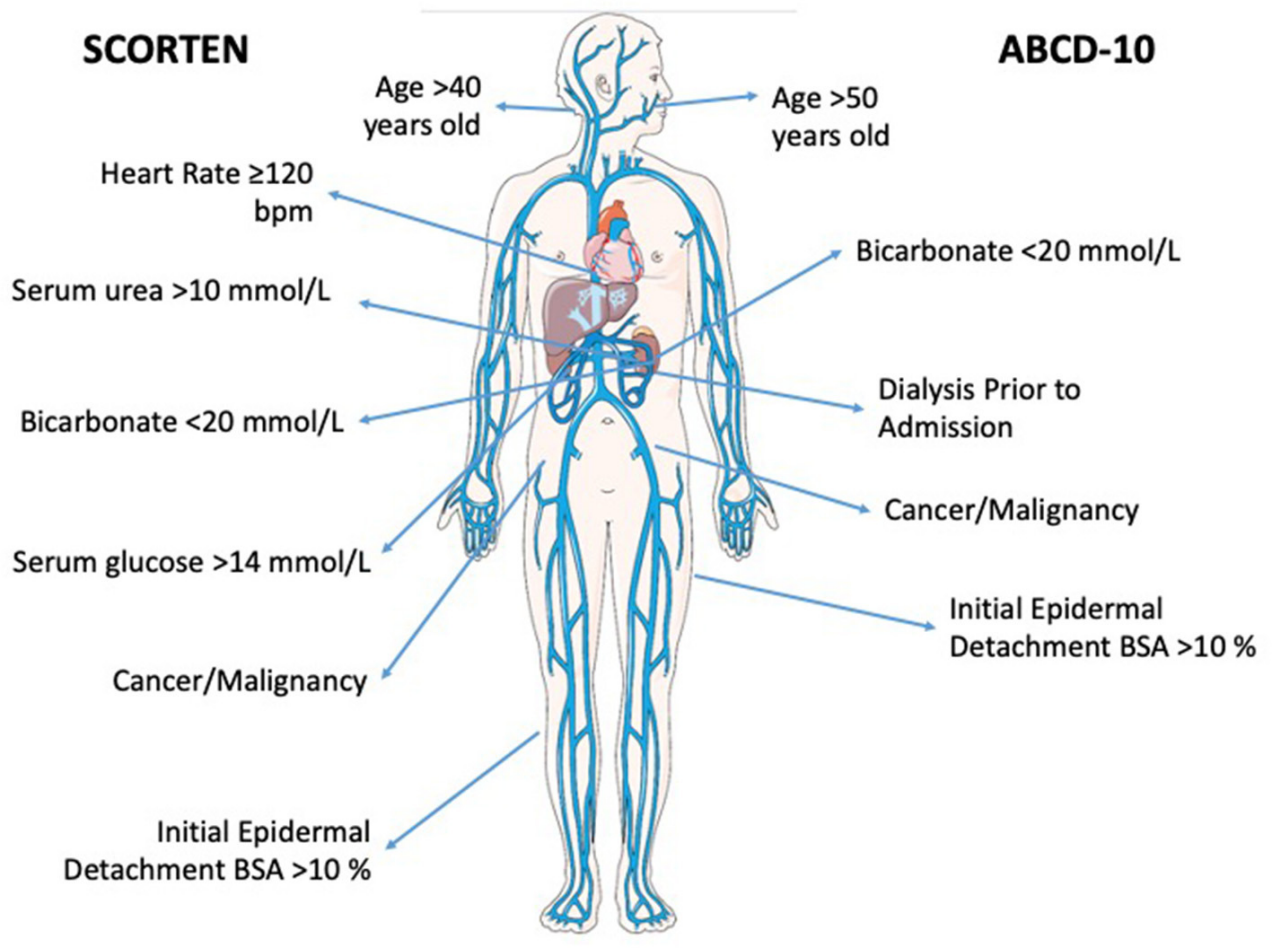
Bioicon representation of the prognostic factors associated with both SCORTEN and ABCD-10 scoring systems. *Venous-circulation-body icon by Servier* *https://smart.servier.com/* *is licensed under CC-BY 3.0 Unported* *https://creativecommons.org/licenses/by/3.0/*.

Another recently devised mortality prognostication tool for epidermal necrolysis is ABCD-10. The ABCD-10 is calculated using the following metrics: age over 50 years (one point), bicarbonate level <20 mmol/L (one point), cancer present and active (two points), dialysis prior to admission (3 points), and epidermal detachment ≥10% body surface area on admission (one point) (Table 1) (13). Despite its recency in development, ABCD-10 offers many strengths when assessing patients with epidermal necrolysis. In comparison to SCORTEN, ABCD-10 takes includes patients with end stage renal disease (using prior dialysis as a proxy) and more heavily weighs cancer diagnosis (Figure 1). Authors of ABCD-10 discovered that undergoing dialysis prior to admission was associated with a more than 15-fold increased risk of death in comparison to those not undergoing dialysis (13). In additional studies since its inception, ABCD-10 has been validated in external cohorts as having good discriminatory capability similar to that of SCORTEN (15). With continuing advances in supportive care and intensive treatments, as well as varying treatment protocols across institutions, ABCD-10 is a great step toward improving prognostic information of epidermal necrolysis patients.

### Comparing SCORTEN vs. ABCD-10

While ABCD-10 has good discriminatory ability, multiple studies have showed that it underperforms in comparison to SCORTEN (3, 7, 15, 16). Specifically, one retrospective cohort study in Singapore found that in both patients treated with supportive care or immunomodulatory therapy, ABCD-10 underestimated mortality at lower score ranges and overestimated mortality at higher score ranges (15). Authors of another large retrospective study in the United States postulated that ABCD-10 underperformed SCORTEN due to the lower rates of dialysis and cancer in their population (3). Furthermore, some researchers have suggested that SCORTEN already adequately captures kidney disease as a co-morbidity by included serum urea and bicarbonate levels, given evidence of multicollinearity between dialysis and serum bicarbonate levels (15).

Further studies are needed to better understand the applicability of ABCD-10. Still, it is limited in its usefulness in epidermal necrolysis assessment, as it cannot be used to monitor cutaneous involvement throughout hospitalization and responsiveness to treatment.

## Clinical Endpoints

While SCORTEN and ABCD-10 are commonly used mortality prognostication tools for epidermal necrolysis, to determine therapeutic efficacy, other clinical endpoints are needed to monitor disease response to interventions. Formal endpoints in clinical trials for patients with epidermal necrolysis have not been standardized. A query of the ClinicalTrials.Gov database for trials evaluating interventions for patients with epidermal necrolysis demonstrated high variability in primary and secondary outcomes (Table 2). Overall, outcomes among clinical trials and retrospective studies are generally grouped into three categories: (1) the standardized mortality ratio, (2) clinical outcomes, and (3) cutaneous response to treatment.

**Table 2:** Endpoints in trials registered at ClinicalTrials.Gov for epidermal necrolysis interventions.

**ClinicalTrials.Gov ID**	**Intervention**	**Primary Outcome**	**Secondary Outcome(s)**
NCT01696500 (17)	Intravenous immunoglobulin (IVIg)	1. Disease evaluation score	1. Disease evaluation score 2. Avulsed skin area 3. Erythematous area
NCT03585946 (18)	Cyclosporine vs. IVIg vs. etanercept vs. steroids	1. Mortality 2. Time to cessation of new lesion formation 3. Time to re-epithelialization 4. Hospital length of stay	
NCT02987257 (19)	Cyclosporine vs. etanercept vs. placebo	1. Time to complete re-epithelialization	1. Time to halting of progression of SJS/TEN skin disease 2. Mortality 3. Actual mortality vs. expected mortality 4. Ocular involvement 5. Infections 6. Hospital length of stay 7. Proportion of patients with adverse events due to assigned treatment arm
NCT02795143 (20)	Isotretinoin vs. supportive care	1. Number of days of hospitalization	1. Percent of body surface area affected
NCT02739295 (21)	G-CSF vs. placebo	1. Time for healing 2. Changes in immunohistologic typing 3. Neutrophilic count	1. WBC count 2. WBC formula
NCT04651439 (22)	G-CSF vs. placebo	1. Arrest of progression at day 5	1. Arrest of progression 2. Complete re-epidermization 3. 30-day survival 4. 1-year survival 5. Duration of hospitalization 6. Premature discontinuation of experimental treatment 7. Adverse events 8. Use of systemic corticosteroid therapy 9. Specialty follow-up 10. Quality of life evolution 11. Risk of developing PTSD
NCT04711200 (23)	Adipose derived stromal cells injected IV	1. Safety: observation of at least one adverse effect 2. Efficacy: rate of complete or almost complete re-epithelialization	1. Rate of observed and predicted death by SCORTEN 2. Duration of hospitalization according to historical cohort related to BSA involved 3. Duration of hospitalization according to historical cohort related to onset of the disease 4. Duration of hospitalization according to historical cohort related to SCORTEN 5. Duration of each mucous membranes healing 6. Rate of sepsis 7. Rate of intensive care transfer 8. Rate of sequelae 9. Th1/Th2 immune response in the peripheral blood of the patients 10. Evaluation of expression profile of Th1/Th2 associated chemokines and anti-inflammatory chemokines in the peripheral blood 11. Epidermal chimerism study on healed skin biopsy 12. Cutaneous re-epithelialization rate

*Inclusion criteria included trials enrolling only patients with a diagnosis of SJS or TEN. Exclusion criteria were trials evaluating only organ specific interventions (e.g., ophthalmologic interventions) or trials that were withdrawn*.

### The Standardized Mortality Ratio

One of the most common primary endpoints utilized in epidermal necrolysis studies is the standardized mortality ratio (SMR), defined as the ratio of observed deaths in comparison to deaths predicted by SCORTEN (13, 24–28). For example, a retrospective cohort analysis on 377 patients across multiple institutions in the United States stratified SMR by therapeutic approach, and demonstrated that combination of intravenous immunoglobulin and steroid use led to the lowest SMR of 0.52 [95% confidence interval (CI) 0.21–0.79] (27). However, the SMR for all patients in this cohort was 0.70 (95% CI 0.58–0.79), suggesting that SCORTEN as a whole overestimated mortality risk in this patient cohort. This has been reflected in other studies that use the SMR (29).

### Clinical Outcomes

Many studies commonly employ basic clinical outcomes, such as length of stay, development of sepsis, and mortality. In a systematic review of the efficacy of intravenous immunoglobulin in the treatment of epidermal necrolysis, clinical endpoints were defined as mortality rates, length of hospital stay, time to disease cessation, and time to skin healing (30). A recent European multicenter study sought to assess overall treatment approaches including supportive care only as the reference group and the treatment groups were systemic glucocorticoids, cyclosporine, intravenous immunoglobulin, and antitumor necrosis factor agents (2). This study classified outcomes as risk of infection, body surface area detachment in the acute phase, and an overall 6-week mortality rate between treatment groups (2). Furthermore, participants were also evaluated for long-term outcomes defined as the development of severe acute complications which included septicemia, acute kidney injury, pulmonary infection, or respiratory distress requiring mechanical ventilation (2). While some of these outcomes are standard clinical outcomes including complicating infections, others are more specific to the disease and lack the validation to confirm their utility such as time to disease cessation, skin healing, and body surface area detachment in the acute phase.

Disease severity is also utilized as an outcome measure, with severity measurements varying between studies. In a study assessing burn unit transfers, disease severity was classified as total body surface area as well as the Acute Physiology and Chronic Health Evaluation (APACHE) score (31). Conversely, other trials utilized their own severity illness scores by developing rating scales which combined lesion characteristics and patient general conditions (32). While these assessments are commonly used for burn and ICU patients, they are of uncertain utility as a primary outcome measure for an intervention to be beneficial.

### Cutaneous Outcome Measures

In addition to mortality and systemic disease severity as primary endpoints, cutaneous signs are an important outcome measure. The most frequently used cutaneous outcomes include time to skin re-epithelialization and body surface area stabilization from the acute phase. However, there are no standardized morphological assessments for cutaneous resolution of the acute phase and therefore, these outcomes are subject to provider bias and unclear validity. Furthermore, these cutaneous endpoints are not sensitive to special site areas such as the mucous membranes. As alluded to previously, subjectivity also arises in grading of BSA involvement. Some studies utilized a cutaneous measure of total BSA of detached and detachable skin (25, 30) that did not include strictly purpuric lesions, while another study defined cutaneous endpoints as the onset of spontaneous resolution of the acute phase (33). Clearly, more discrete skin scoring assessments and instruments are necessary to be validated for the success of future clinical studies in this disease. Further, improved cutaneous scoring assessments are critical not only as an outcome measure, but as an entry criterion for research studies to ensure balanced randomization across institutions.

## Conclusion

The lack of standardized endpoint measures in epidermal necrolysis is a significant barrier in the development of regulatory approved therapies. At the current time, there exists a panoply of drugs, wound care, and supportive care regimens that lack strong evidence for efficacy for treating this disease. Efforts to improve treatment options and reduce mortality require standardized clinical outcomes that are more finely tuned to risk-stratifying patients at entry, then detecting treatment response. Recently some there have been some attempts at standardization of quantitative endpoints via a survey that identified minimally clinical important differences (MCID), defined as the smallest change in a treatment outcome that a patient or clinician would identify as important and indicate a change in management (34).

Further work is required on standardizing outcome measures and validating skin assessments. We recommend the development of a consensus morphological assessment of cutaneous morphologies and locations of involvement, from which cutaneous endpoints can be reliably measured. Without these standardizations, therapeutic treatments and interventions will remain limited with a bias toward lack of intervention efficacy.

## Author Contributions

AD, SH, MW, and BK contributed to the writing of the manuscript. AD and BK prepared the final manuscript. All authors contributed to the article and approved the submitted version.

## Conflict of Interest

The authors declare that the research was conducted in the absence of any commercial or financial relationships that could be construed as a potential conflict of interest.

## Publisher's Note

All claims expressed in this article are solely those of the authors and do not necessarily represent those of their affiliated organizations, or those of the publisher, the editors and the reviewers. Any product that may be evaluated in this article, or claim that may be made by its manufacturer, is not guaranteed or endorsed by the publisher.
